# Challenging neonatal central nervous system fungal infection diagnosed by MRI: a case report

**DOI:** 10.3389/fped.2025.1691551

**Published:** 2025-12-15

**Authors:** Hanin Shatrit, Salahaldeen Deeb, Motaz Tamimi, Mohammad I. Smerat, Ibrahim Alzatari

**Affiliations:** 1 Faculty of Medicine, Al-Quds University, Jerusalem, Palestine; 2 Department of Pediatrics Neurology, Hebron University, Hebron, Palestine; 3 Department of Radiology, Al Ahli Hospital, Hebron, Palestine

**Keywords:** central nervous system, fungal infection, magnetic resonance imaging, neonate, magnetic resonance imaging (MRI)

## Abstract

**Background:**

Although central nervous system fungal infection (CNSF) is uncommon, it is a life-threatening condition that requires prompt diagnosis and management. Here, we report a full-term female neonate with suspected CNSF diagnosed by magnetic resonance imaging (MRI) despite repeatedly negative cerebrospinal fluid (CSF) and blood cultures, in whom the causative pathogen could not be identified.

**Case presentation:**

A full-term female neonate presented on the first day of life with irritability, hypoactivity, cyanosis, poor feeding, and two episodes of generalized tonic-clonic seizures with up-rolling of the eyes and lip smacking. Workup for neonatal sepsis showed negative CSF and blood cultures. MRI findings suggested CNSF, and treatment was initiated with low-dose conventional amphotericin B deoxycholate (0.5 mg/kg/day) for 4 weeks, leading to complete clinical and radiological resolution.

**Conclusion:**

Negative CSF and blood cultures do not exclude neonatal CNSF. In resource-limited settings, MRI findings together with clinical response to antifungal therapy may support a diagnosis of probable CNSF, even when the pathogen is not identified and standard high-dose regimens are not available.

## 1 Background

Central nervous system fungal infection (CNSF) is rare in children, especially during the neonatal period. According to the literature, the true incidence of CNSF is unknown, although the prevalence of cryptococcal meningitis is estimated to be nearly one per 100,000 (1). To the best of our knowledge, 34 cases of CNSF during the neonatal period have been reported. The most dangerous and potentially fatal complication of an invasive fungal infection is dissemination to the central nervous system (CNS) via hematogenous spread, which is the most common route, or direct extension; the related fatality rates surpass 90% (2). CNSF is a neurological emergency that may be localized or diffuse, involving the meninges, brain, and spinal cord (3). Since it may be lethal, CNSF requires a precise and prompt diagnosis to minimize potential consequences, such as poor neurodevelopmental outcomes.

## 2 Case presentation

We report a female infant who was delivered via uncomplicated normal vaginal delivery at term with a birth weight of 3 kg to a 19-year-old gravida 1 para 1 mother with an uneventful pregnancy and normal fetal ultrasound. On the first day of life, she presented with irritability, hypoactivity, cyanosis, poor feeding, and two episodes of generalized tonic-clonic seizures with up-rolling of the eyes and lip smacking, and was admitted to the neonatal intensive care unit (NICU) of a regional referral hospital in Hebron, Palestine. On examination, her temperature was 36.9 °C; she had a full bulging fontanel, weak primitive reflexes, reactive and symmetrical pupils with horizontal nystagmus, axial and limb hypotonia with normal spontaneous antigravity movements, and brisk (grade 3+) deep tendon reflexes. Growth parameters, including length, weight, and head circumference, were within normal limits.

On investigation, the neonate was hypoglycemic, so a 10% dextrose bolus was administered and the hypoglycemia was resolved. Complete blood count showed a white blood cell count of 14,000/mm^3^. In addition, she was found to have hyperammonemia, elevated blood urea nitrogen levels, abnormal liver function tests [elevated aspartate transaminase/alanine transaminase levels, hypoalbuminemia, and prolonged prothrombin time], and prolonged activated partial thromboplastin time, all of which returned to normal levels before discharge. Serological testing resulted in elevated cytomegalovirus (CMV) and toxoplasma immunoglobulin G (IgG) titers with normal immunoglobulin M (IgM) titers for both pathogens. A lumbar puncture showed normal values with negative culture results. A blood culture and urinalysis were also negative.

Additional fungal diagnostics, such as serum or cerebrospinal fluid (CSF) β-D-glucan, mannan antigen, cryptococcal antigen, and fungal polymerase chain reaction (PCR) or metagenomic next-generation sequencing (mNGS) were not available in our hospital and therefore could not be performed.

**Figure 1:**
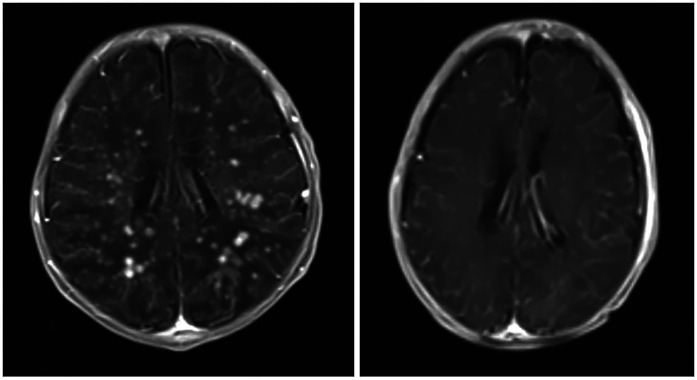
Axial views of post-contrast MRI show the difference between before (right) and after (left) treatment. The enhancing nodules have dramatically disappeared, indicating significant improvement. The previously seen meningeal enhancement is also improved, with several residual hyperintense T2 foci in the posterior periventricular region. There is also a suggestion of several tiny susceptibility-weighted imaging hypointense foci in the posterior periventricular region (sites of previous nodules), which may represent areas of calcification. This could be confirmed by a follow-up brain CT.

**Figure 2:**
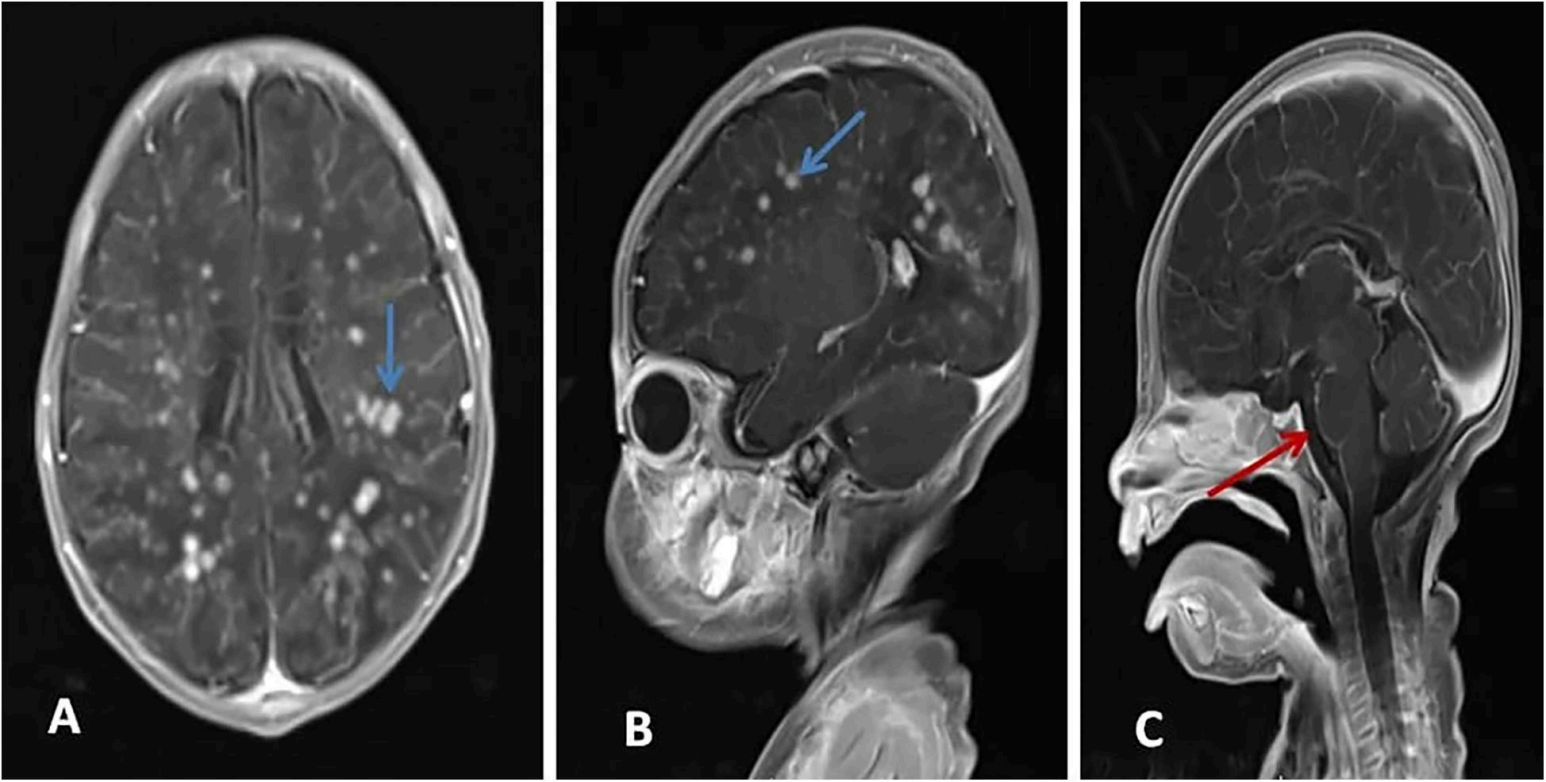
Axial **(A)** and sagittal **(B,C)** views of post-contrast MRI show avid nodular enhancement (blue arrows) and leptomeningeal enhancement (Red arrows).

**Figure 3:**
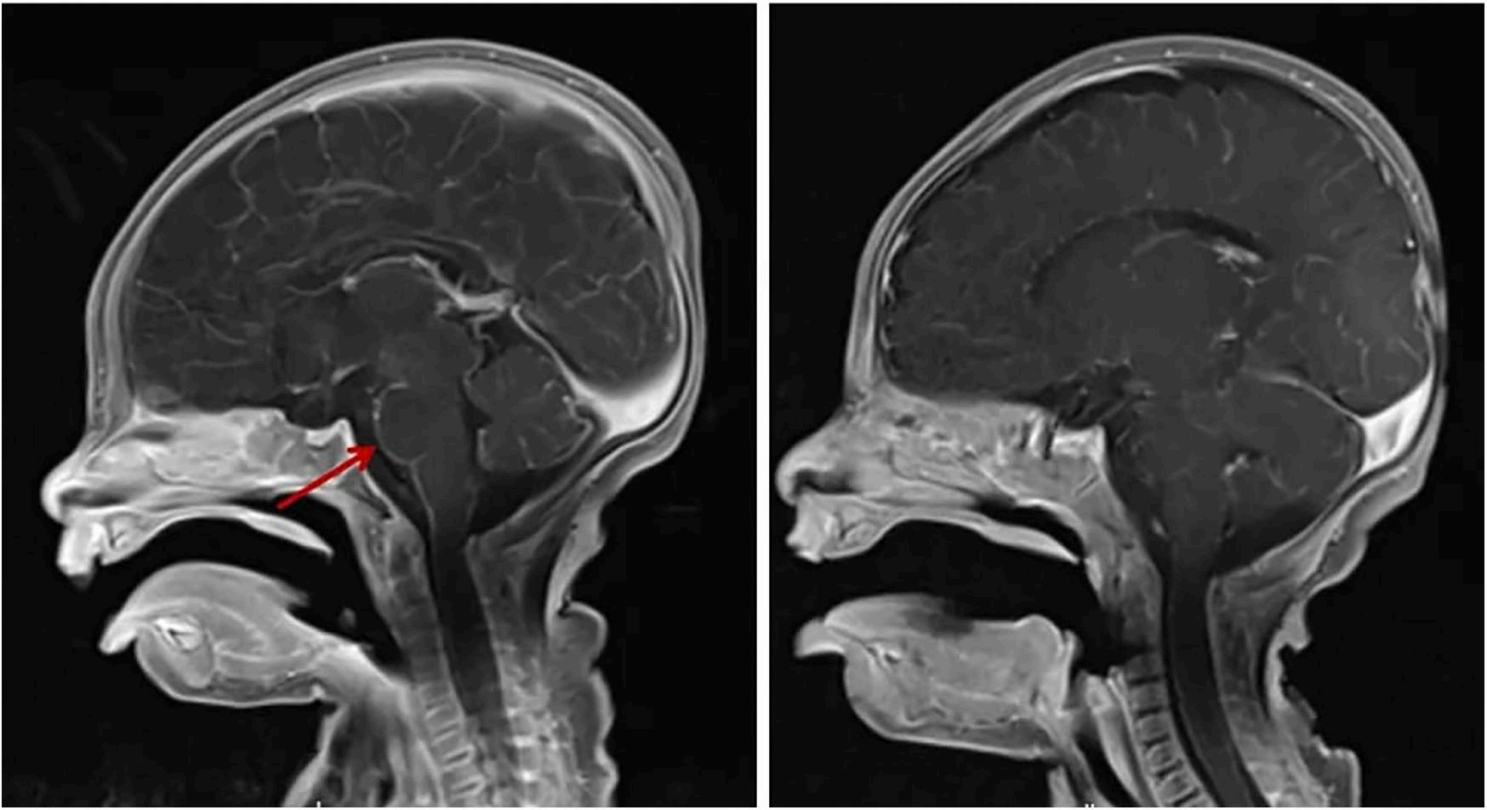
Sagittal views of post-contrast MRI show the difference between before (right) and after (left) treatment. The previously seen nodular and meningeal enhancements are improved.

Magnetic resonance imaging (MRI) of the brain with and without contrast, performed in the Department of Radiology at Al Ahli Hospital, Hebron, Palestine, showed numerous small, faintly hyperintense fluid-attenuated inversion recovery (FLAIR) foci scattered in both cerebral hemispheres’ subcortical and periventricular white matter. Each was less than 5 mm in size, with avid nodular enhancement. Additionally, there was diffuse thin supratentorial leptomeningeal enhancement and infratentorial meningeal enhancement surrounding the brainstem and cerebellum, without edema. Cerebrospinal fluid spaces appeared normal, and there were no congenital abnormalities. The conclusion suggested multiple disseminated supratentorial small enhancing nodules with leptomeningeal enhancement, raising suspicion of viral or fungal infection (Figures 1–3).

Based on these MRI findings, an infectious disease consultation was obtained and conventional amphotericin B deoxycholate was started at 0.5 mg/kg/day due to suspicion of CNSF. Therapy was continued for 4 weeks. Because of concerns about nephrotoxicity and the lack of liposomal amphotericin B or flucytosine in our unit, we used a conventional amphotericin B deoxycholate dose of 0.5 mg/kg/day, which is lower than the doses (around 1.0 mg/kg/day) recommended in the current Infectious Diseases Society of America (IDSA) guidelines for neonatal CNS candidiasis. This resource-adapted regimen should not be considered standard therapy in high-resource settings. A repeated MRI 4 weeks later was clear, with complete resolution of the previously noted enhancing nodules and meningeal enhancement, and the infant's clinical symptoms had completely resolved. She was discharged home with advice to continue normal breastfeeding and to attend regular follow-up with a neurologist.

During the 4-week course of amphotericin B, serum creatinine, potassium, magnesium, and alanine aminotransferase were monitored at least twice weekly and remained within the local neonatal reference ranges, and no clinically significant nephrotoxicity, electrolyte disturbances, or hepatotoxicity were observed.

## 3 Discussion

CNSF in neonates remains an uncommon but potentially fatal condition, with high reported mortality in very-low-birth-weight infants despite treatment (4, 5). Most described cases occur in preterm infants or those with recognizable predisposing factors such as prolonged hospitalization, invasive devices, parenteral nutrition, abdominal or neurosurgical procedures, prolonged broad-spectrum antibiotics, or immunodeficiency (6, 7). The present case is noteworthy because the infant was full-term, had none of these conventional risk factors, and yet developed MRI-confirmed CNSF with complete clinical response to antifungal therapy. This highlights that neonatal CNSF can occur even in the absence of recognized risk factors and that early imaging may be crucial in such atypical presentations.

Neonatal *Candida* infections remain a significant cause of morbidity in intensive care settings, particularly among hospitalized infants exposed to invasive procedures or prolonged antibiotic therapy. Previous multicenter studies have demonstrated rising incidences of neonatal candidiasis and highlighted its association with substantial mortality, especially in preterm and low-birth-weight populations (8, 9). Surveillance data from NICUs further emphasize that *Candida* species account for a considerable proportion of late-onset sepsis, with outcomes closely linked to the timeliness of diagnosis and antifungal initiation (10). Likewise, colonization studies across neonatal units have shown that systemic infection often follows gastrointestinal or skin colonization, underscoring the need for vigilance, even in infants without overt risk factors (11).

Although maternal colonization or nosocomial exposure may contribute to neonatal fungal acquisition, none were identifiable in this case. Subtle or transient neonatal immune immaturity may also predispose a neonate to invasive fungal infection, even in otherwise healthy infants, though this is generally difficult to document clinically. Importantly, repeatedly negative blood and CSF cultures do not exclude CNS fungal disease, as demonstrated in several studies of neonatal and pediatric candidiasis (12–14). Candidemia is frequently absent in *Candida* meningitis, and culture sensitivity is further reduced when blood volumes are limited, as is common in newborns.

### 3.1 Diagnostic challenges and the limitations of CSF analysis

The non-specific clinical features of CNSF, including irritability, seizures, apnea, respiratory distress, and feeding difficulties, overlap extensively with bacterial meningitis and metabolic or hypoxic conditions, complicating early recognition (15, 16). The present infant displayed irritability, seizures, feeding problems, and abnormal tone features consistent with invasive CNS infection but not specific to fungal etiology.

CSF indices are notoriously unreliable in neonatal *Candida* meningitis. Cohen-Wolkowiez et al. reported that nearly half of neonates with culture-confirmed *Candida* meningitis had normal CSF glucose, protein, and leukocyte counts (17). Similarly, Faix et al. found abnormal CSF leukocytosis in only 20%–52% of cases (6, 18). These observations reinforce that normal CSF values do not exclude CNS candidiasis and justify adjunctive diagnostic modalities, particularly neuroimaging. Furthermore, delayed CSF sample processing may reduce leukocytes by up to 50% within 1 h (19), which may have contributed to the normal CSF profile in this case.

Advanced fungal diagnostics recommended in high-resource settings, such as serum or CSF β-D-glucan, mannan antigen, cryptococcal antigen, PCR, or mNGS, were unavailable in our hospital. This constraint prevented etiological confirmation and limits diagnostic certainty, but it also reflects the reality of many neonatal units globally. We explicitly acknowledge that the diagnosis in this case should be considered probable rather than proven, consistent with IDSA definitions for invasive candidiasis.

### 3.2 Role of MRI and comparison with differential diagnoses

Neuroimaging, particularly MRI, is a critical tool for detecting occult CNSF and differentiating it from other infectious and non-infectious etiologies (20). MRI in this neonate demonstrated numerous small enhancing FLAIR nodules with diffuse leptomeningeal enhancement, which subsequently resolved after antifungal therapy. Similar MRI-based diagnoses have been reported in culture-negative neonatal fungal infections (Table 1), although most cases involve preterm infants or older neonates (21–23).

**Table 1:** Reported neonatal CNS fungal cases diagnosed by MRI (culture-negative).

Author (year)	Patient age	MRI findings	Final diagnosis (organism)	Treatment
Mehta et al. (21)	20 days	Multiple cerebral abscesses (bilateral)	*Candida albicans*	Burr-hole drainage + 6 wk antifungal therapy
Oh et al. (22)	Neonate (days old)	Multiple intracerebral abscesses (by MRI)	*Aspergillus* (brain abscess)	Amphotericin B + flucytosine + itraconazole
Chae et al. (23)	30 week preterm (1,426 g)	Numerous small disseminated hyperintense lesions in the bilateral white matter and basal ganglia	Presumed fungal (yeast) brain abscesses	Liposomal amphotericin B + fluconazole
Our case	1-day-old, full-term	Numerous small enhancing FLAIR nodules with diffuse leptomeningeal enhancement	Suspected Candida CNS infection	Amphotericin B for 4 weeks; full recovery

Wk, week, MRI, magnetic resonance imaging.

Multiple small enhancing lesions in neonatal white matter generate a wide differential diagnosis. Key distinguishing points include the following. Pyogenic bacterial cerebritis or microabscesses typically produce diffusion-restriction foci with greater vasogenic edema, mass effect, and coalescing ring-enhancing abscesses, which were features not seen in this case (24).

Furthermore, mycobacterial or parasitic granulomas often show thicker, irregular enhancement, basal cistern predilection, or calcification on follow-up imaging (24, 25); none were present in our patient.

Moreover, TORCH infections, particularly CMV and toxoplasmosis, usually exhibit robust calcifications, ventriculomegaly, malformations, or diffuse white-matter injury (26), all of which were absent in this case.

Hypoxic ischemic encephalopathy and metabolic disorders cause symmetrical watershed or deep gray nuclei involvement and linear or gyriform enhancement rather than innumerable punctate nodules (27).

Finally, tumor-like infiltrative processes (e.g., congenital leukemia, high-grade glioma) generally produce mass effect, hemorrhage, or a dominant lesion; none of which were present in this case (27).

The infant's radiologic pattern of innumerable tiny enhancing white-matter nodules, mild leptomeningeal enhancement, absence of calcifications or mass effect, and complete resolution after antifungal therapy is most consistent with disseminated fungal microabscesses, in line with reported MRI patterns of neonatal *Candida* CNS disease (25, 28). Although organism-specific radiologic signatures are not definitive, the distribution and enhancement pattern most closely match candidiasis rather than cryptococcal pseudocysts, *Aspergillus* hemorrhagic infarcts, or endemic dimorphic fungal infections (29).

### 3.3 Therapeutic considerations and resource-based constraints

According to the IDSA guidelines, the preferred regimen for neonatal *Candida* meningitis is amphotericin B (ideally liposomal) plus flucytosine (30, 31). Flucytosine is favored because of its superior CSF penetration; however, it is often avoided in neonates due to bone marrow and hepatic toxicity (32). In our setting, neither liposomal amphotericin B nor flucytosine was available. Thus, conventional amphotericin B deoxycholate at 0.5 mg/kg/day was used, a lower dose than the ∼1.0 mg/kg/day recommended internationally.

We clearly emphasize that this reduced-dose monotherapy was resource-driven and should not be considered standard of care. Despite this, the infant demonstrated full clinical recovery and complete radiologic resolution after 4 weeks of therapy. Serial monitoring of creatinine, electrolytes, and liver enzymes revealed no significant toxicities. This favorable outcome reinforces prior observational studies showing that amphotericin B monotherapy, even at lower doses, may be beneficial in early, localized, or less severe neonatal CNS candidiasis when alternative agents are unavailable.

As summarized in Table 1, previously reported neonatal CNS fungal infections diagnosed by MRI have mainly involved older or preterm infants, typically presenting with multiple cerebral or intracerebral abscesses and requiring prolonged antifungal therapy, often with surgical intervention in *Candida albicans* and *Aspergillus* cases (21–23). Our case is distinct in representing a full-term neonate, only 1 day old, with innumerable small enhancing FLAIR nodules and diffuse leptomeningeal enhancement, managed successfully with amphotericin B alone and complete radiological and clinical recovery.

This report has several important limitations. First, the diagnosis of CNSF in our infant remains probable rather than proven because all blood and CSF cultures were negative and advanced fungal investigations (β-D-glucan, mannan antigen, cryptococcal antigen, fungal PCR, and mNGS) were not available in our setting. Second, quantitative MRI metrics, such as apparent diffusion coefficient values and signal-intensity ratios, were not systematically recorded, and imaging interpretation was based on consensus review rather than formal inter-reader agreement statistics. Third, we did not perform formal standardized neurodevelopmental testing (e.g., Bayley-III), so long-term neurocognitive outcomes remain uncertain. Finally, our observations derive from a single-center, resource-limited neonatal unit without access to liposomal amphotericin B or flucytosine, which limits the generalizability of our findings to high-resource settings.

This case report provides valuable clinical insight due to several notable strengths. First, it documents an exceptionally rare presentation of CNSF in a full-term neonate with no identifiable risk factors and repeatedly negative blood and CSF cultures, underscoring an atypical but clinically important scenario. The diagnosis and management were guided primarily by MRI findings, demonstrating the crucial diagnostic role of neuroimaging in culture-negative CNSF and offering a practical framework for clinicians in similar settings. Comprehensive pre- and post-treatment MRI scans allowed for objective assessment of therapeutic response, supporting the utility of imaging as a surrogate endpoint when laboratory confirmation is unavailable. The successful management of this infant involved coordinated collaboration among neonatology, pediatric neurology, radiology, and infectious disease specialists, reflecting multidisciplinary clinical practice standards. Finally, the availability of the raw clinical and imaging data upon reasonable request enhances transparency and promotes external validation, further strengthening the scientific value of this report.

## 4 Conclusion

In summary, CNSF is rare, particularly in neonates. This study suggests that MRI can be a useful diagnostic method for suspected CNSF in cases with negative CSF and blood culture results, in association with a high suspicion of CNSF based on the clinical background. In addition, the clinical improvement and the resolution of MRI lesions in response to antifungal treatment could be supportive for the diagnosis.

## Data Availability

The datasets presented in this article are not readily available because of ethical and privacy restrictions. Requests to access the datasets should be directed to the corresponding author.
